# Comparison between optical coherence tomography-guided and intravascular ultrasound-guided primary percutaneous coronary intervention for ST-segment elevation myocardial infarction

**DOI:** 10.20407/fmj.2023-006

**Published:** 2023-11-29

**Authors:** Yuji Matsuwaki, Takashi Muramatsu, Yukio Ozaki, Takashi Uwatoko, Takuo Toriya, Hidemaro Takatsu, Yu Yoshiki, Masataka Yoshinaga, Masato Ishikawa, Masaya Ohota, Hideaki Ota, Hideo Izawa

**Affiliations:** 1 Department of Cardiology, Cardiovascular Center, Fujita Health University Hospital, Toyoake, Aichi, Japan; 2 Department of Cardiology, Fujita Health University Okazaki Medical Center, Okazaki, Aichi, Japan

**Keywords:** Coronary artery disease, Myocardial infarction, Percutaneous coronary intervention, Intravascular ultrasound, Optical coherence tomography

## Abstract

**Objective:**

To examine the clinical outcomes of optical coherence tomography (OCT)-guided percutaneous coronary intervention (PCI) in patients presenting with ST-segment elevation myocardial infarction (STEMI).

**Methods:**

We retrospectively investigated 533 consecutive patients who underwent primary PCI for STEMI between June 2016 and December 2020. The primary endpoint was a target lesion failure (TLF; defined as a composite of cardiac death, target vessel myocardial infarction, or target lesion revascularization). Propensity score (PS) matching was performed to allow direct comparison of OCT-guided and intravascular ultrasound (IVUS)-guided PCI.

**Results:**

Patients in the OCT group (n=166) were younger than those in the IVUS group (n=367) and had a significantly higher left ventricular ejection fraction and estimated glomerular filtration rate. Killip class IV and left main stem disease were more common in the IVUS group. The median peak creatine kinase level was comparable between the two groups (1953 U/L vs 1603 U/L). A significantly larger amount of contrast was used in the OCT group (200 mL vs 165 mL; p<0.001). The cumulative incidence of TLF during a median follow-up of 2.2 years did not differ significantly between OCT and IVUS groups (9.6% vs 13.6%; p=0.221) but cardiac mortality was significantly higher in the IVUS group (8.7% vs 3.6%; p=0.047). After PS matching (n=161 in each group), there was no significant between-group difference in TLF or any other clinical outcome measures.

**Conclusions:**

OCT-guided PCI demonstrated clinical outcomes in patients with STEMI that were comparable to those of IVUS-guided PCI despite considerable differences in background characteristics.

## Introduction

The clinical outcome in patients presenting with ST-segment elevation myocardial infarction (STEMI) has improved considerably in recent decades.^1^ Primary percutaneous coronary intervention (PCI) is perceived to be a major factor contributing to the reduction in mortality,^2,3^ and more than 40,000 cases presenting with STEMI undergo primary PCI annually in Japan.^4^ Intravascular ultrasound (IVUS) has been recognized as an important intracoronary imaging technique, and recent studies suggest that a greater reduction in the risk of major adverse cardiac events can be achieved by IVUS-guided PCI than by conventional PCI under angiographic guidance alone.^5–7^ Specifically, the incidences of target vessel revascularization and definite stent thrombosis were found to be significantly lower in patients presenting with STEMI who underwent IVUS-guided PCI than in those who underwent angio-guided PCI despite no difference in all-cause mortality; however, the difference disappeared after adjustment for confounding factors.^8^

The newer light-based intracoronary imaging techniques, such as optical coherence tomography (OCT) and optical frequency domain imaging (OFDI), have an advantage in terms of a higher spatial resolution in comparison with IVUS. This advantage allows for better detection of not only an etiology but also procedural complications in patients with acute coronary syndrome (ACS).^9,10^ Randomized trials demonstrated that OCT/OFDI-guided PCI was not inferior to IVUS-guided PCI in terms of clinical outcomes or imaging surrogates in selected patients with chronic coronary syndrome.^11–13^ However, there are still limited data on OCT/OFDI-guided PCI specifically in patients with STEMI. The aim of this study was to compare the characteristics and clinical outcomes of OCT-guided PCI with those of IVUS-guided PCI in patients presenting with STEMI.

## Methods

### Study design and population

The study had a retrospective, single-center, observational design and included patients who presented with STEMI within 12 h of symptom onset and underwent primary PCI at Fujita Health University Hospital (Toyoake, Japan) between June 2016 and December 2020. Patients in whom neither OCT/OFDI nor IVUS was performed (i.e., angiographic guidance alone was used) and those in whom both OCT/OFDI and IVUS were used during the primary PCI procedure were excluded. Both OCT and OFDI were included in the OCT group for the purposes of analysis.

### PCI procedure and intravascular imaging protocol

Primary PCI was performed via the radial, femoral, or brachial artery using 6–8 Fr guiding catheters. Selection of vascular access, guide wires, balloon catheters, stents, and other interventional devices was left to the discretion of the operators. All patients received intravenous injections of unfractionated heparin (100 U/kg) and a nonionic low-osmolality contrast agent (i.e., iohexol or iomeprol). All patients were loaded with 200 mg of aspirin and 300 mg of clopidogrel or 20 mg of prasugrel (a specific dose in Japan) orally if not already given. PCI was performed using the standard technique and use of intracoronary imaging was left to the discretion of the operators in accordance with the following specific criteria for use of OCT/OFDI at our institution: stable hemodynamics without cardiogenic shock, congestive heart failure, or other conditions requiring infusion of inotropes and/or mechanical circulatory support; Thrombolysis in Myocardial Infarction flow grade ≥2 at time of acquisition of OCT images; preserved renal function with an estimated glomerular filtration rate (eGFR) ≥45 mL/min/1.73 m^2^ or no known history of chronic kidney disease; non aorto-ostial lesions; and absence of angiographic findings suggestive of spontaneous coronary artery dissection. OCT/OFDI and IVUS imaging procedures were performed using dedicated consoles (Ilumien Optis^®^, Abbott Vascular, Santa Clara, CA, USA; Lunawave^®^; Terumo Corporation, Tokyo, Japan; and Visiwave^®^ or Visicube^®^, Terumo) and imaging catheters (Dragonfly Optis^®^, Abbott Vascular; Fast View^®^, Terumo; and ViewIT^®^ or AltaView^®^, Terumo). For acquisition of OCT/OFDI images, 8–12 mL of 100% contrast agent were injected manually. The protocol used for intracoronary imaging guidance during the procedure is described elsewhere (Supplementary Materials).^13^ Drug-coated balloons were used for small vessels or in-stent restenosis at the discretion of the operators.

### Clinical follow-up

All patients received dual antiplatelet therapy consisting of aspirin (100 mg) plus clopidogrel (75 mg) or prasugrel (3.75 mg) for 6–12 months after primary PCI based on the 2018 guideline published by the Japanese Circulation Society.^14^ The duration of dual antiplatelet therapy was limited to a maximum of 1 month or generally to the hospital stay after primary PCI for patients on anticoagulant therapy. After discharge from hospital, clinical follow-up consisted of regular visits or telephone contact. Follow-up angiography was performed at 8–12 months after the primary PCI procedure. Otherwise, patients underwent coronary computed tomography angiography or stress myocardial perfusion imaging.

### Clinical outcome measures

The primary outcome was a target lesion failure (TLF), which was defined as a composite of cardiac death, target vessel myocardial infarction (i.e., re-infarction), or clinically-driven target lesion revascularization. Secondary endpoints were all-cause death, spontaneous myocardial infarction, and definite stent thrombosis. Definitions of all these outcome measures have been described elsewhere.^15^

### Statistical analysis

Continuous variables are expressed as the median and interquartile range (IQR) and categorical variables as the number and percentage. Continuous variables were compared between the study groups using the Mann–Whitney *U* test and categorical variables using the chi-squared test or Fisher’s exact test. Cumulative event rates were estimated using the Kaplan–Meier method, and differences therein were determined using the log-rank test. In view of significant differences in baseline characteristics between the groups, a logistic regression model was used to develop a propensity score (PS) using five independent variables among the baseline characteristics, including age, left ventricular ejection fraction (LVEF), hemoglobin, eGFR, Killip class IV, presence of a left main lesion, and use of an intra-aortic balloon pump (IABP) to allow direct comparisons. After adjustment using the PS with a 1:1 matching method, we generated a matched cohort of patients who underwent OCT/OFDI-guided PCI or IVUS-guided PCI. Cox regression models were applied to identify predictors of the primary outcome on a patient-level basis. The multivariable model was created using a forced entry method, whereby the independent variables were removed at the 0.01 significance level considering the number of events. If the variables were highly correlated with each other (r>0.5 and p<0.05), those that had a higher level of significance were eligible for inclusion in the multivariable model.

All statistical analyses were performed using SPSS software version 28.0 (IBM Corp., Armonk, NY, USA). All tests were two-sided, and a p-value <0.05 was considered statistically significant.

## Results

**Figure 1:**
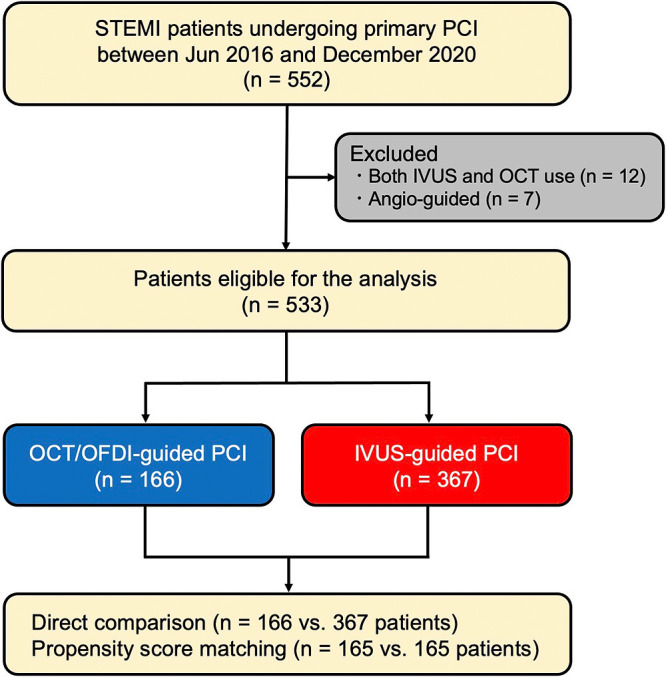
Flow diagram showing the patient selection process for this study.

Figure 1 shows the patient selection process as a flow diagram. A total of 552 patients with STEMI underwent primary PCI between June 2016 and December 2020. After exclusion of 12 patients in whom both OCT/OFDI and IVUS were performed during the primary PCI procedure and 7 patients who underwent angio-guided PCI, data for 166 patients who underwent OCT/OFDI-guided PCI (31.1%) and 367 who underwent IVUS-guided PCI (68.9%) were available for direct comparisons (i.e., the crude population). After adjustment using the PS matching method, 161 patients in each group were included for matched comparisons (i.e., the matched population).

### Background data

The patient demographics are shown in Table 1. In comparison with the IVUS group, the OCT group was younger and had a higher LVEF and eGFR. The proportions of patients with Killip class IV and left main stem disease were higher in the IVUS group than in the OCT group; however, the peak creatine kinase level was comparable between the study groups. Prasugrel and a renin-angiotensin system inhibitor (i.e., an angiotensin-converting enzyme inhibitor or angiotensin receptor blocker) were prescribed more frequently and direct oral anticoagulants less frequently at discharge in the OCT group than in the IVUS group. After PS matching, all demographic variables were well balanced between the two groups.

Lesion and procedural characteristics are shown in Table 2. Radial access and manual aspiration thrombectomy were more common in the OCT group than in the IVUS group. A majority of patients (90.2%) were treated with drug-eluting stents (DES); the stents used were longer in the IVUS group than in the OCT group but there was no significant between-group difference in stent diameter. The amount of contrast volume was significantly higher in the OCT group than in the IVUS group (200 mL vs 165 mL; p<0.001). Mechanical circulatory support (i.e., IABP and veno-arterial extracorporeal membrane oxygenation) were used more frequently in the IVUS group than in the OCT group. Significant differences in the frequency of manual aspiration thrombectomy and amount of contrast volume remained even after the PS matching.

### Clinical outcomes

**Figure 2:**
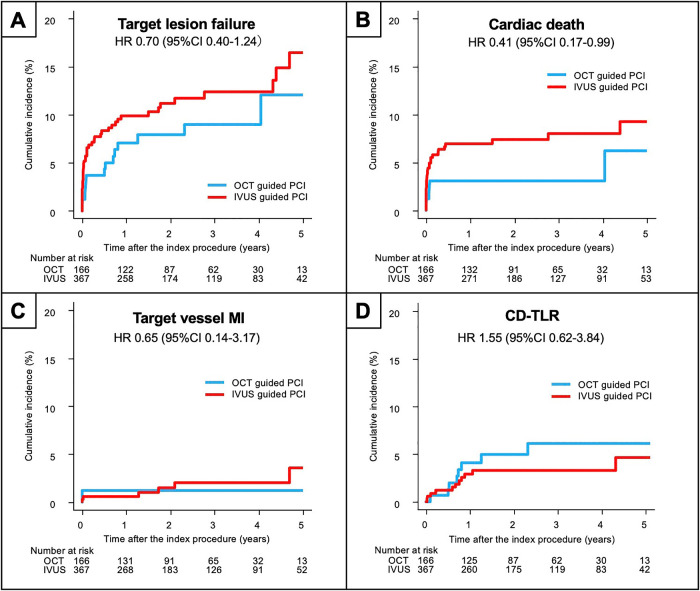
Kaplan–Meier curves for the clinical outcomes in the crude population. (A) Primary composite endpoint. (B) Cardiac death. (C) Target-vessel myocardial infarction. (D) Clinically-driven target lesion revascularization. IVUS, intravascular ultrasound; OCT, optical coherence tomography

**Figure 3:**
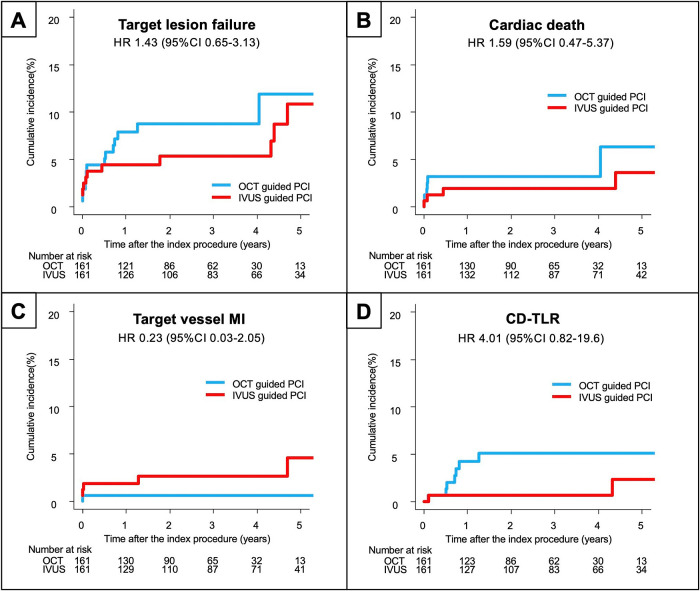
Kaplan–Meier curves for the clinical outcomes in the matched population (A) Primary composite endpoint. (B) Cardiac death. (C) Target vessel myocardial infarction. (D) Clinically-driven target lesion revascularization. IVUS, intravascular ultrasound; OCT, optical coherence tomography

The clinical outcomes are summarized in Table 3. The median follow-up duration was 2.2 years (IQR 1.0, 3.9). There was no significant difference in the cumulative incidence of the primary outcome (i.e., TLF) between the OCT group and the IVUS group (9.6% vs 13.6%; p=0.221), whereas the cardiac mortality rate was significantly higher in the IVUS group (8.7% vs 3.6%; p=0.047). Kaplan–Meier estimates revealed no significant difference in the incidence of the composite TLF (p=0.218), target vessel myocardial infarction (p=0.595), or TLR (p=0.346) between the two groups; however, the incidence of cardiac death was significantly higher in the IVUS group than in the OCT group (p=0.040, Figure 2). In the matched population, the median follow-up duration was 2.9 years (IQR 1.3, 4.5). There was no significant difference in the primary outcome measure between the two groups (Figure 3). The results were similar after excluding patients with a left main stem lesion, those in whom an IABP was used, and those with Killip class IV (Supplementary Materials).

### Predictors of the primary outcome

Multivariable logistic regression analysis identified LVEF, Killip class IV, and the left main coronary artery as the culprit vessel to be independent predictors of the primary outcome and that OCT-guided PCI was not (Table 4).

## Discussion

To the best of our knowledge, this study is the first to compare the clinical outcomes of IVUS-guided PCI with those of OCT-guided PCI exclusively in patients presenting with STEMI. Our main findings were as follows: 1) there were considerable differences in baseline characteristics between the patients treated under OCT guidance and those treated under IVUS guidance; 2) there was no significant between-group difference in the cumulative incidence of TLF, although cardiac mortality was significantly higher in the IVUS group; and 3) there was no significant difference in any clinical outcome measure after adjustment by PS matching.

Recent guidelines in Western countries^16,17^ and the Japanese expert consensus document^3^ recommend primary PCI using the newer-generation DES for treatment of patients presenting with STEMI. Although no clear evidence has been established, intracoronary imaging techniques have been widely used in these patients in Japan. Unlike angiography, intracoronary imaging provides morphologic information concerning the atherosclerotic plaque responsible for MI. The etiology of coronary thrombosis (e.g., plaque rupture, plaque erosion, or calcified nodule) can be better identified by OCT/OFDI because its spatial resolution is higher than that of IVUS. This advantage of OCT/OFDI may alter risk stratification and the management of future adverse cardiac events in patients experiencing ACS.^18^ Another major role of intracoronary imaging is to optimize stent implantation with achievement of the following targets: better stent expansion, avoidance of the landing zone when the plaque burden is >50% or the tissue is lipid-rich, and avoidance of largely malapposed regions, irregular tissue protrusion, and major dissections.^19^ The ULTIMATE trial demonstrated that the risk of target vessel failure (i.e., cardiac death, target vessel myocardial infarction, or clinically-driven target vessel revascularization) at 12 months was significantly lower in patients who underwent IVUS-guided PCI than in those who underwent angio-guided PCI (2.9% vs 4.2%, hazard ratio 0.53). Moreover, in the IVUS-guided PCI group, the incidence of target vessel failure was significantly lower in patients with optimal stent implantation than in those with suboptimal stent implantation (1.6% vs 4.4%, hazard ratio 0.35).^20^ Although no randomized controlled trial has investigated intracoronary imaging guidance in the setting of STEMI, a nationwide database study in the US and a recent registry study in the UK have reported a steady increase in the number of intracoronary imaging procedures performed in patients presenting with ACS.^21,22^

In the present study, 98.7% of patients were treated under the guidance of intracoronary imaging devices because they are fully reimbursed in Japan. IVUS was used more frequently than OCT/OFDI (68.9% vs 31.1%). A possible explanation for this finding is our use of strict criteria for OCT/OFDI such that patients with unstable hemodynamics, those with Thrombolysis in Myocardial Infarction flow grade <2 at the time of image acquisition, and those with impaired renal function are excluded. Consequently, in the crude population, proportions with Killip class IV and left main stem disease were higher and eGFR was lower in the IVUS group than in the OCT group. Furthermore, patients in the IVUS group had a significantly lower LVEF and received mechanical circulatory support, such as IABP and veno-arterial extracorporeal membrane oxygenation, more often than the OCT group. Our data show that the incidence of cardiac death was significantly lower in the OCT group than in the IVUS group, but this finding disappeared in the matched population. Our finding of no significant difference in the primary outcome between the OCT/OFDI-guided PCI group and the IVUS-guided PCI group is consistent with the results of the OPINION study, which was the first randomized controlled trial powered to detect clinical outcomes of target vessel failure within 12 months and showed that OCT-guided PCI was non-inferior to IVUS-guided PCI in patients with chronic coronary syndrome.^12^ Therefore, the difference in clinical outcomes probably reflects a difference in patient characteristics or hemodynamic conditions rather than type of intracoronary imaging device used.

A potential drawback of OCT/OFDI is the need to remove blood from the lumen during image acquisition. It could be argued that IVUS is a preferable approach to OCT/OFDI considering that the contrast volume has been positively associated with the risk of contrast-induced nephropathy (CIN) in patients with ACS undergoing emergent PCI.^23^ James et al. reported that CIN was associated with increased risks of mortality, cardiovascular events, and prolonged hospitalization.^24^ Our data indeed showed that the contrast volume was significantly greater in the OCT group than in the IVUS group in both the crude and matched populations. This finding is in line with the results of previous trials comparing OFDI-guided and IVUS-guided PCI.^12,13^ However, when the KDIGO definition was used,^25^ the incidence of CIN was significantly lower in the OCT group than in the IVUS group for the crude population (8.4% vs 18.8%, p=0.002) but not for the matched population (8.6% vs 14.8%, p=0.119). Furthermore, the contrast volume was not a predictor of the primary outcome (i.e., TLF) in logistic regression analysis. These findings suggest that patient comorbidities or hemodynamic status rather than contrast volume might contribute to the incidence of CIN and clinical outcomes.

The main strength of this study is that it investigated real-world data. However, it also has several limitations. First, it had a retrospective, single-center, observational design. Therefore, the generalizability of our results needs further elucidation. Second, we did not compare our results with those obtained by conventional PCI under angiographic guidance because of the unique reimbursement of intracoronary imaging devices in Japan. Third, we applied pre-specified criteria for use of OCT and the imaging guidance protocol, which means that it might be challenging to standardize the PCI procedure in the setting of STEMI. Finally, although every effort was made to adjust for patient characteristics for the purposes of a PS score-matched analysis, the possibility of unmeasured confounders and selection bias cannot be excluded. Therefore, our present results can only be considered hypothesis-generating.

In conclusion, OCT-guided PCI showed clinical outcomes comparable to those of IVUS-guided PCI in patients presenting with STEMI despite considerable between-group differences in background characteristics and hemodynamic status. Larger studies and randomized controlled trials are needed to clarify the clinical advantages of OCT-guided PCI over IVUS-guided PCI in the setting of STEMI.

## Figures and Tables

**Table1:** Patient characteristics

	Crude population				Matched population		
	OCT group (n=166)	IVUS group (n=367)	p-value		OCT group (n=161)	IVUS group (n=161)	p-value
Age, years	68.0 (59.0, 75.0)	71.0 (61.5, 79.0)	0.008		68.0 (59.0, 75.0)	68.0 (59.0, 76.0)	0.817
Male sex, n (%)	123 (74.1)	290 (79.0)	0.219		120 (74.1)	120 (74.1)	1.000
Body mass index, kg/m^2^	23.3 (21.4, 25.4)	23.7 (21.4, 26.0)	0.375		23.3 (21.2, 25.3)	23.7 (21.8, 25.9)	0.258
Hypertension, n (%)	99 (59.6)	242 (65.9)	0.173		96 (59.6)	108 (67.1)	0.203
Diabetes mellitus, n (%)	41 (24.7)	115 (31.3)	0.124		39 (24.2)	48 (29.8)	0.315
Dyslipidemia, n (%)	96 (57.8)	182 (49.6)	0.092		92 (57.1)	89 (55.3)	0.822
Current smoker, n (%)	54 (32.5)	111 (30.2)	0.614		53 (32.9)	55 (34.2)	0.725
Prior MI, n (%)	17 (10.2)	31 (8.4)	0.516		16 (9.9)	14 (8.7)	0.848
Prior PCI, n (%)	23 (13.9)	41 (11.2)	0.390		22 (13.3)	20 (12.3)	0.869
Prior CABG, n (%)	0	4 (1.1)	0.177		0	3 (1.9)	0.248
Hemodialysis, n (%)	4 (2.4)	6 (1.6)	0.511		4 (2.4)	3 (1.9)	1.000
LVEF, %	50 (45, 53)	47 (39, 55)	0.042		48 (42, 55)	47 (42, 55)	0.765
Hemoglobin, g/dL	12.8 (11.6, 14.3)	13.0 (11.7, 14.4)	0.728		12.8 (11.6, 14.4)	13.5 (12.0, 14.8)	0.133
LDL cholesterol, mg/dL	109 (88, 128)	108 (86, 132)	0.923		109 (89, 128)	114 (88, 134)	0.273
eGFR, mL/min/1.73 m^2^	76.9 (63.0, 88.7)	70.0 (51.5, 86.7)	0.003		76.8 (63.0, 88.0)	77.0 (64.0, 93.8)	0.617
Peak creatine kinase, U/L	1953 (835, 3104)	1603 (737, 3504)	0.700		1853 (782, 3096)	1600 (712, 3162)	0.491
Killip class Ⅳ, n (%)	3 (1.8)	29 (7.9)	0.005		3 (1.9)	0	0.248
Medication at discharge, n (%)							
Aspirin	166 (100.0)	359 (97.8)	0.062		161 (100.0)	160 (99.4)	1.000
Clopidogrel	10 (6.0)	36 (9.8)	0.150		9 (5.6)	13 (8.1)	0.508
Prasugrel	153 (92.2)	313 (85.3)	0.026		149 (92.5)	141 (87.6)	0.192
RAS inhibitor	136 (81.9)	259 (70.6)	0.006		132 (82.0)	123 (76.4)	0.272
Β-blocker	135 (81.3)	271 (73.8)	0.060		130 (80.7)	122 (75.8)	0.344
Statin	163 (98.2)	351 (95.6)	0.141		158 (98.1)	153 (95.0)	0.219
Vitamin K antagonist	4 (2.4)	14 (3.8)	0.406		4 (2.5)	6 (3.7)	0.750
Direct oral anticoagulant	3 (1.8)	22 (6.0)	0.034		3 (1.9)	7 (4.3)	0.336

CABG, coronary artery bypass graft; eGFR, estimated glomerular filtration rate; IVUS, intravascular ultrasound; LDL, low-density lipoprotein; LVEF, left ventricular ejection fraction; MI, myocardial infarction; OCT, optical coherence tomography; PCI, percutaneous coronary intervention; RAS. renin-angiotensin system

**Table2:** Lesion and procedural characteristics

	Crude population				Matched population		
	OCT group (n=166)	IVUS group (n=367)	p-value		OCT group (n=161)	IVUS group (n=161)	p-value
Diseased vessels, n (%)			0.538				0.715
One-vessel disease	98 (59.0)	205 (55.9)			96 (59.6)	96 (59.6)	
Two-vessel disease	49 (29.5)	107 (29.2)			47 (29.2)	51 (31.7)	
Three-vessel disease	19 (11.4)	55 (15.0)			18 (11.1)	14 (8.7)	
Left main stem disease	1 (0.6)	29 (7.9)	<0.001		1 (0.6)	1 (0.6)	1.000
Culprit vessel, n (%)			0.063				0.105
Right coronary artery	64 (38.6)	149 (40.6)			62 (38.5)	61 (37.9)	
Left anterior descending	89 (53.6)	169 (6.0)			86 (53.4)	75 (46.6)	
Left circumflex artery	13 (7.8)	39 (10.7)			13 (8.1)	25 (15.6)	
Left main coronary artery	0	10 (2.7)			0	0	
PCI procedural characteristics							
Radial approach, n (%)	135 (75.3)	245 (66.8)	0.033		122 (75.8)	113 (70.2)	0.315
Stent diameter, mm	3.0 (2.75, 3.5)	3.0 (3.0, 3.5)	0.120		3.0 (2.75, 3.5)	3.0 (2.9, 3.5)	0.458
Stent length, mm	22.0 (15.5, 28.0)	24.0 (18.0, 33.0)	<0.001		22.0 (15.0, 28.0)	23.0 (18.0, 29.0)	0.137
Drug-eluting stent, n (%)	155 (93.4)	326 (88.8)	0.116		152 (94.4)	145 (90.1)	0.211
Drug-coated balloon, n (%)	10 (6.0)	22 (6.0)	1.000		9 (5.6)	7 (4.3)	0.799
Thrombus aspiration, n (%)	138 (83.1)	213 (58.0)	<0.001		132 (82.0)	103 (64.0)	<0.001
Final TIMI grade 3, n (%)	160 (96.4)	336 (91.6)	0.044		155 (96.3)	154 (95.1)	0.598
Door-to-balloon time, min	85 (70, 90)	89 (87, 98)	<0.001		85 (69, 90)	87 (68, 90)	0.502
Procedure time, min	81 (63, 93)	90 (75, 107)	<0.001		81 (63, 93)	90 (71, 114)	0.001
Contrast volume, mL	200 (170, 239)	165 (138, 165)	<0.001		200 (170, 235)	163 (140, 204)	<0.001
Mechanical circulatory support, n (%)							
IABP	11 (6.6)	98 (26.7)	<0.001		10 (6.2)	11 (6.8)	1.000
Impella	2 (1.2)	14 (3.8)	0.167		2 (1.2)	0	0.498
VA-ECMO	0	16 (4.4)	0.004		0	0	NA

IABP, intra-aortic balloon pump; IVUS, intravascular ultrasound; OCT, optical coherence tomography; TIMI, Thrombolysis in Myocardial Infarction; VA-ECMO, veno-arterial extracorporeal membrane oxygenation

**Table3:** Clinical outcomes

Variable, n (%)	Crude population					Matched population			
	OCT group (n=166)	IVUS group (n=367)	HR (95% CI)	p-value		OCT group (n=161)	IVUS group (n=161)	HR (95% CI)	p-value
Target lesion failure	16 (9.6)	50 (13.6)	0.70 (0.40–1.24)	0.221		14 (8.7)	12 (7.5)	1.43 (0.65–3.13)	0.377
Cardiac death	6 (3.6)	32 (8.7)	0.41 (0.17–0.99)	0.047		6 (3.7)	5 (3.1)	1.59 (0.47–5.37)	0.457
Target vessel MI	2 (1.2)	7 (1.9)	0.65 (0.14–3.17)	0.597		1 (0.6)	5 (3.1)	0.23 (0.03–2.05)	0.190
CD-TLR	8 (4.8)	11 (3.0)	1.55 (0.62–3.84)	0.350		7 (4.3)	2 (1.2)	4.01 (0.82–19.6)	0.090
All-cause death	17 (10.2)	55 (17.0)	0.66 (0.39–1.14)	0.140		16 (9.9)	17 (10.6)	1.16 (0.58–2.31)	1.000
All MI	4 (2.4)	17 (4.6)	0.41 (0.12–1.40)	0.153		3 (1.9)	15 (9.3)	0.21 (0.06–7.14)	0.013
Definite ST	1 (0.6)	1 (0.3)	2.21 (0.14–35.35)	0.575		1 (0.6)	0	N/A	N/A

CA-AKI, contrast-associated acute kidney injury; CD-TLR, clinically-driven target lesion revascularization; CI, confidence interval; HR, hazard ratio; IVUS, intravascular ultrasound; MI, myocardial infarction; OCT, optical coherence tomography; ST, stent thrombosis

**Table4:** Factors identified to predict the primary outcome as a composite of cardiac death, target-vessel myocardial infarction, and clinically-driven target lesion revascularization

Variable	Univariable analysis				Multivariable analysis		
	HR	95%CI	p-value		HR	95%CI	p-value
Age (years)	1.04	1.01–1.06	0.004		1.02	0.99–1.05	0.082
Male sex	1.31	0.70–2.45	0.402				
Body mass index (kg/m^2^)	0.88	0.82–0.95	0.001		0.96	0.89–1.03	0.282
Hypertension	0.97	0.59–1.61	0.910				
Diabetes mellitus	1.67	1.02–2.75	0.042				
Dyslipidemia	0.88	0.54–1.44	0.613				
Current smoker	0.55	0.30–1.00	0.052				
Prior MI	1.39	0.66–2.91	0.387				
Prior PCI	1.17	0.58–2.36	0.666				
Prior CABG	4.21	1.02–17.34	0.047				
Hemodialysis	2.92	0.91–9.31	0.070				
LVEF (%)	0.94	0.92–0.95	<0.001		0.97	0.95–0.99	0.013
Hemoglobin (g/dL)	0.87	0.78–0.98	0.022				
LDL cholesterol (mg/dL)	0.98	0.98–0.99	0.002		0.99	0.99–1.00	0.338
eGFR (mL/min/1.73 m^2^)	0.98	0.97–0.99	<0.001		0.99	0.98–1.00	0.098
Killip class Ⅳ	8.18	4.62–14.47	<0.001		3.66	1.74–7.70	<0.001
Culprit vessel							
Right coronary artery	0.72	0.43–1.21	0.212				
Left anterior descending	0.81	0.50–1.33	0.406				
Left circumflex	1.42	0.70–2.86	0.334				
Left main coronary artery	9.37	4.89–18.00	<0.001		3.63	1.72–7.68	<0.001
Radial approach	0.49	0.30–0.80	0.004		0.80	0.48–1.34	0.392
Contrast volume (mL)	1.00	0.99–1.00	0.814				
OCT/OFDI-guided PCI	0.72	0.41–1.26	0.251		1.23	0.67–2.26	0.501

CABG, coronary artery bypass graft; CI, confidence interval; eGFR, estimated glomerular filtration rate; HR, hazard ratio; LDL, low-density lipoprotein; LVEF, left ventricular ejection fraction; MI, myocardial infarction; OCT, optical coherence tomography; OFDI, optical frequency domain imaging; PCI, percutaneous coronary intervention

## Declarations

### Availability of data and materials

The datasets used during this study are available from the corresponding author upon reasonable request.
