# Supplementary material for: Comparison between optical coherence tomography-guided and intravascular ultrasound-guided primary percutaneous coronary intervention for ST-segment elevation myocardial infarction
Source: Fujita Med J. 2023 Nov 29;10(1):16–23. doi: 10.20407/fmj.2023-006 (PMC10847631; doi:10.20407/fmj.2023-006)
Supplement: Supplementary file 1 — Supplementary Materials [file fmj-10-016-s001.pdf]

Procedural protocol for OCT/OFDI-guided and IVUS-guided PCI [1]

	OCT/OFDI-guided PCI	IVUS-guided PCI
**1st. Pre-dilatation**	• Pre-dilatation with a 2.0-mm semi-compliant balloon is allowed only when catheter passage through the lesion was unsuccessful or blood removal was insufficient potentially resulting in poor image quality for analyses.<br>• Not specified for subsequent pre-dilatation after imaging investigation.	• Pre-dilatation with a 2.0-mm semi-compliant balloon is allowed only when catheter passage through the lesion was unsuccessful.<br>• Not specified for subsequent pre-dilatation after imaging investigation.
**2nd. Stent landing zone (i.e. reference sites)**	• Ideally the most normal looking sites that had the largest lumen without lipidic plaque >2 quadrants or suggestive thin-cap fibroatheroma (TCFA).<br>• Length will be measured between the proximal and the distal reference sites.	• Ideally the most normal looking sites that had the largest lumen with a percentage plaque area of ≤50 %.<br>• Length will be measured between the proximal and the distal reference sites.
**3rd. Stent sizing**	• By measuring lumen area at the proximal and the distal reference sites.<br>• Nominal size will be selected by taking 10 % or 0.25-mm larger than mean lumen diameter.	• By measuring vessel area (external elastic lamina) at the proximal and the distal reference sites.<br>• In case with diseased reference sites (e.g. diffuse lesions), lumen diameter can also be referred.
**4th. Post-dilatation**	• A non-compliant balloon with a nominal diameter 0.25-mm larger than that of selected stent is recommended.	• Not specified
**5th. Stent optimization**	• Stent expansion index ≥80 %<br>• Malapposed stent strut <20 %<br>• Absence of major edge dissection	

OCT = optical coherence tomography; OFDI = optical frequency domain imaging; IVUS = intravascular ultrasound; PCI = percutaneous coronary intervention

Comparison between OCT and IVUS in the crude population after excluding the patients with left main lesion, IABP use, or Killip IV

Table S1. Patient characteristics

	OCT group (n=153)	IVUS group (n=248)	p value
Age years	68.0 (59.0, 75.0)	71.0 (60.0, 78.3)	0.035
Male gender, n (%)	113 (73.9)	191 (77.0)	0.474
Body mass index, kg/m^2	23.3 (21.3, 25.5)	24.0 (21.9, 25.9)	0.320
Hypertension, n (%)	89 (58.2)	164 (66.1)	0.112
Diabetes mellitus, n (%)	36 (23.5)	69 (27.8)	0.353
Dyslipidemia, n (%)	88 (57.5)	131 (52.8)	0.409
Current smoker, n (%)	52 (34.0)	73 (29.4)	0.375
Prior MI, n (%)	14 (9.2)	18 (7.3)	0.570
Prior PCI, n (%)	20 (13.1)	26 (10.5)	0.426
Prior CABG, n (%)	0 (0.0)	3 (1.9)	0.290
Hemodialysis, n (%)	4 (2.4)	4 (1.6)	0.487
LVEF, %	49 (44, 55)	51 (44, 55)	0.332
Hemoglobin, g/dL	12.8 (11.6, 14.3)	13.0 (11.9, 14.4)	0.608
LDL cholesterol, mg/dL	106 (87, 128)	111 (90, 132)	0.305
eGFR, mL/kg/m^2	76.8 (63.0, 88.0)	77.0 (64.0, 93.8)	0.617
Peak creatine kinase, U/L	1860 (768, 2954)	1312 (542, 2779)	0.125
Killip class IV, n (%)	0 (0.0)	0 (0.0)	NA
Medication at discharge, n (%)			
Aspirin	153 (100.0)	246 (99.2)	0.527
Clopidogrel	8 (5.2)	18 (7.3)	0.533
Prasugrel	142 (92.8)	222 (89.5)	0.292
RAS inhibitors	130 (85.0)	193 (77.8)	0.092
B-blocker	123 (80.4)	179 (72.2)	0.074
Statin	151 (98.7)	240 (96.8)	0.330

Vitamin K antagonist	4 (2.6)	8 (3.2)	1.000
Direct oral anticoagulants	2 (1.3)	11 (4.4)	0.144

OCT = optical coherence tomography; IVUS = intravascular ultrasound; MI = myocardial infarction; PCI = percutaneous coronary intervention; CABG = coronary artery bypass graft; LVEF = left ventricular ejection fraction; LDL = low density lipoprotein; eGFR = estimated glomerular filtration rate; RAS = renin-angiotensin system

Table S2. Lesion and procedural characteristics

	OCT group (n=153)	IVUS group (n=248)	p value
Number of diseased vessels, n (%)			0.407
1 vessel disease	91 (59.5)	150 (60.5)	
2 vessel disease	43 (28.1)	77 (31.0)	
3 vessel disease	19 (12.4)	21 (8.5)	
Left main disease	0 (0.0)	0 (0.0)	NA
Culprit vessel, n (%)			0.208
Right coronary artery	59 (38.6)	103 (41.5)	
Left anterior descending	83 (54.2)	116 (46.8)	
Left circumflex	11 (7.2)	29 (11.7)	
Left main coronary artery	0 (0.0)	0 (0.0)	NA
PCI procedural characteristics			
Radial approach, n (%)	117 (76.5)	180 (72.6)	0.413
Stent diameter, mm	3.00 (2.75, 3.50)	3.0 (3.00, 3.50)	0.258
Stent length, mm	22.0 (15.0, 28.0)	24.0 (18.0, 33.0)	0.001
Drug eluting stent, n (%)	142 (92.8)	225 (90.7)	0.581
Drug coated balloon, n (%)	10 (6.5)	13 (5.2)	0.660
Thrombus aspiration, n (%)	126 (82.4)	146 (58.9)	<0.001
Final TIMI grade 3, n (%)	148 (96.7)	243 (98.0)	0.515
Door-to-Balloon time, min	83 (69, 90)	89 (87, 98)	<0.001
Procedure time, min	80 (63, 93)	89 (70, 105)	0.001
Contrast volume, mL	200 (170, 235)	160 (130, 200)	<0.001
Mechanical circulatory support, n (%)			
IABP	0 (0.0)	0 (0.0)	NA
Impella	0 (0.0)	0 (0.0)	NA
VA-ECMO	0 (0.0)	0 (0.0)	NA

OCT = optical coherence tomography; IVUS = intravascular ultrasound; TIMI = thrombolysis in myocardial infarction; IABP = intra-aortic balloon pumping; VA-ECMO = veno-arterial extracorporeal membrane oxygenation

Table S3. Clinical outcomes

	OCT group (n=153)	IVUS group (n=248)	Hazard Ratio (95% CI)	P value
Target lesion failure, n (%)	14 (9.2)	16 (6.5)	1.49 (0.72-3.05)	0.281
Cardiac death, n (%)	4 (2.6)	8 (3.2)	0.87 (0.26-2.92)	0.827
Target vessel MI, n (%)	2 (1.3)	2 (0.8)	1.63 (0.23-11.59)	0.624
CD-TLR, n (%)	8 (5.2)	6 (2.4)	2.21 (0.77-6.38)	0.142
All cause death, n (%)	14 (9.2)	24 (9.7)	0.97 (0.50-1.88)	0.930
All MI, n (%)	4 (2.6)	7 (2.8)	0.70 (0.18-2.70)	0.602
Definite ST, n (%)	1 (0.6)	0 (0.0)	N/A	N/A

OCT = optical coherence tomography; IVUS = intravascular ultrasound; MI = myocardial infarction; CD-TLR = clinically-driven target lesion revascularization; ST = stent thrombosis; CIN = contrast induced neuropathy

Figure S1. Kaplan-Meyer curves for the clinical outcomes

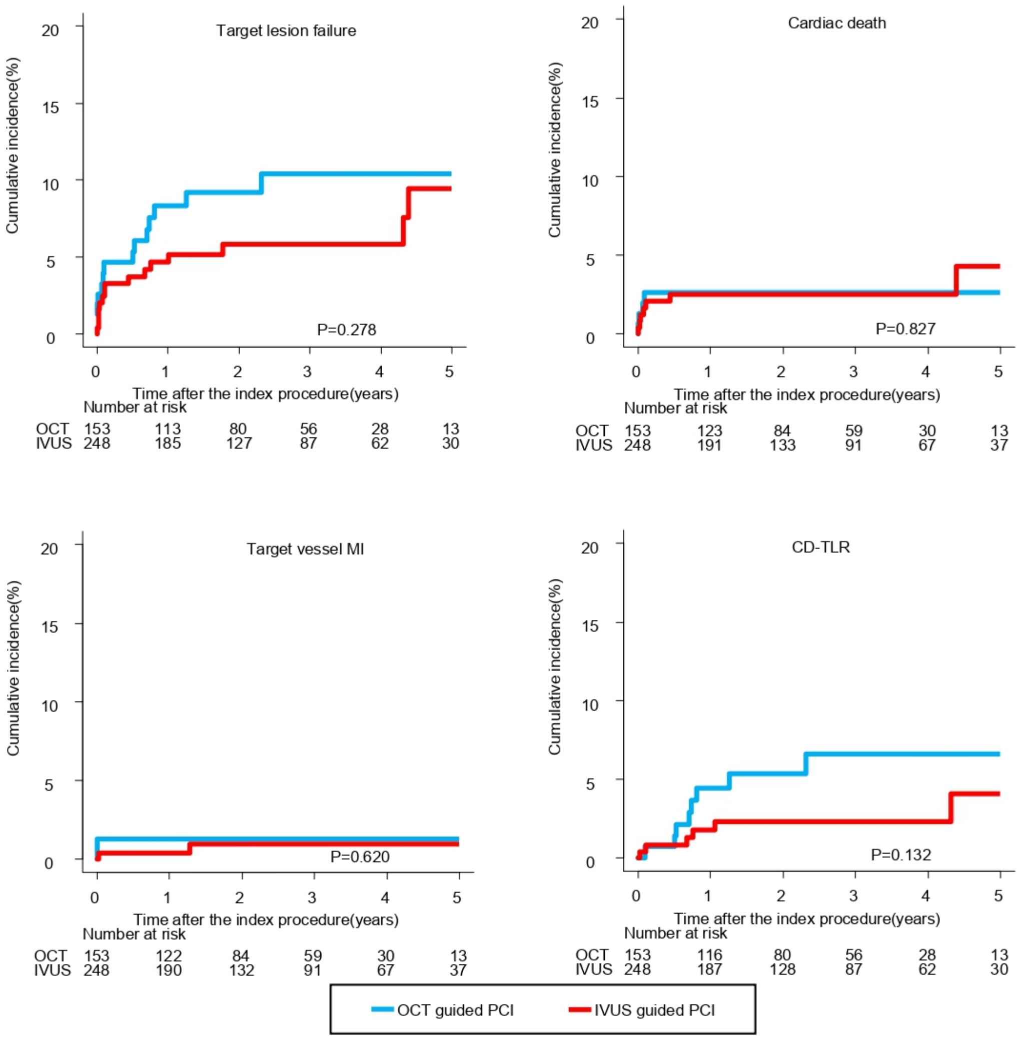

References

1. Muramatsu T, Ozaki Y, Nanasato M, Ishikawa M, Nagasaka R, Ohota M, Hashimoto Y, Yoshiki Y, Takatsu H, Ito K, Kamiya H, Yoshida Y, Murohara T, Izawa H, Investigators M-. Comparison Between Optical Frequency Domain Imaging and Intravascular Ultrasound for Percutaneous Coronary Intervention Guidance in Biolimus A9-Eluting Stent Implantation: A Randomized MISTIC-1 Non-Inferiority Trial. Circ Cardiovasc Interv 2020; 13: e009314.
